# Targeting colorectal cancer with hesperidin and hesperetin: a comprehensive review of antiproliferative activity, chemopreventive, anti-inflammatory, and antioxidant effects

**DOI:** 10.3389/fnut.2025.1647977

**Published:** 2025-12-03

**Authors:** Ming Qi, Yu Su

**Affiliations:** 1 Department of Breast and Thyroid Surgery, Shandong Provincial Hospital Affiliated to Shandong First Medical University, Jinan, Shandong, China; 2 Hangzhou Heyunjia Hospital, Hangzhou, Zhejiang, China

**Keywords:** colorectal cancer, hesperidin, apoptotic, anti-inflammatory, antioxidant

## Abstract

Colorectal cancer (CRC) residues one of the leading reasons of cancer-related mortality worldwide, with increasing incidence attributed to dietary, environmental, and genetic factors. Despite advances in conventional therapies, including chemotherapy and targeted agents, treatment resistance and adverse side effects highlight the need for novel, safer, and more effective therapeutic strategies. Hesperidin (HSD) and hesperetin (HST), bioflavonoid abundantly found in citrus fruits, has emerged as a promising candidate due to its wide-ranging biological activities, particularly its role in modulating key molecular pathways involved in carcinogenesis. This comprehensive review explores the multifaceted anticancer potential of HSD and HST in the context of CRC, focusing on its ability to control programmed cell death mechanisms like autophagy and apoptosis, suppress chronic inflammation, and counteract oxidative stress—three pivotal hallmarks of tumor initiation and progression. Additionally, we examine the synergistic effects of HSD and HST with standard chemotherapeutic agents and its potential as a chemopreventive or adjuvant therapeutic compound. By consolidating preclinical and emerging clinical evidence, this review highlights the translational value of HSD and HST in CRC prevention and management. Understanding its molecular underpinnings may pave the way for HSD- and HST-based nutraceuticals and targeted therapies, contributing to a more personalized and integrative approach to CRC treatment.

## Introduction

Colorectal cancer (CRC) manifests within the entirety or segments of the large intestine, situated at the terminal region of the digestive tract, encompassing a diverse array of neoplastic entities, including numerous carcinoma tumors, lymphomas, sarcomas, and gastrointestinal stromal tumors. The initial presentations are characterized by the emergence of benign polyps, which comprise different adenomas, hyperplastic polyps, sessile serrated polyps, inflammatory polyps, and prevalent serrated adenomas, frequently occurring without overt signs. Nevertheless, these lesions can be identified via screening procedures, which is the rationale behind the recommendation by healthcare practitioners for screening subjects over the age of 50 (1, 2). The non-specific clinical manifestations associated with this malignancy may encompass hematochezia, alterations in bowel habits, unintentional weight loss, and persistent fatigue, necessitating a meticulous differential diagnosis facilitated by biopsy through colonoscopy or sigmoidoscopy. Important risk determinants for CRC encompass advancing biological sex, age, obesity, excessive consumption of dietary sugars and fats, alcohol intake, ingestion of red meats and processed food items, and insufficient physical activity levels. An additional salient factor is the presence of a familial history of colorectal carcinoma, wherein the likelihood of developing this malignancy escalates two to threefold for individuals with a lineage predisposed to the disease (3, 4). In a global context, more than 1 million individuals receive a diagnosis of CRC annually. It ranks as the second greatest predominant neoplasm among females and the third greatest prevalent neoplasm among males, thereby establishing itself as the fourth leading reason of mortality attributable to cancer, following lung, gastric, and hepatic cancers (5, 6).

Hesperidin (HSD) is as a flavanone glycoside that is mainly located in citrus fruits. Its aglycone counterpart is referred to as hesperetin (HST). The initial isolation of HSD from citrus peel was accomplished by the French chemist Lebreton. Due to its diverse array of biological actions, HSD is frequently categorized as a bioflavonoid. HSD is classified as a β-7-rutinoside of HST because it comprises an aglycone, namely HST, and a disaccharide known as rutinose. Both HSD and HST, have demonstrated a variety of biological activities (7, 8). For instance, HSD exhibits vitamin-like properties and has the capacity to reduce capillary permeability, leakiness, and fragility. It additionally demonstrated properties that are anticarcinogenic, anti-inflammatory, antioxidant, and antiallergic (7). A substantial corpus of research has emerged since that time, detailing its novel pharmacological effects, mechanisms of action, and molecular targets. For instance, the implications of HST and HSD on the central nervous system have emerged as a focal point of investigation over the preceding decade, a subject that had not been previously examined (9, 10). Recent discoveries further indicated that the antioxidant effects of HSD extend beyond mere radical scavenging, as it also enhances cellular antioxidant defenses through the ERK/Nrf2 signaling pathway (11, 12).

Emerging evidence from *in vivo, in vitro*, and nanotechnology-based studies highlights the potential of HSD and HST to modulate important molecular pathways involved in CRC pathogenesis, like inflammatory responses, oxidative stress, apoptosis, and cell cycle regulation (13–15). However, these findings are scattered across various experimental models and require a unified and critical synthesis to better understand their therapeutic potential and translational relevance. The objective of present study is to assess a comprehensive and up-to-date review of the antiproliferative, chemopreventive, anti-inflammatory, and antioxidant impacts of HST and HSD in the context of CRC. This review seeks to synthesize findings from *in vivo* and *in vitro* studies, elucidate the underlying molecular mechanisms, assess their potential in several stages of CRC, and explore advancements in drug delivery strategies to enhance their therapeutic efficacy. The ultimate goal is to evaluate the potential of HSD and HST as adjunctive agents for the treatment and prevention of CRC.

## Antiproliferative activity of HSD and HST

The proliferation of cancerous cells is contingent upon two critical cellular characteristics, which encompass the augmentation of cellular division and the suppression or deactivation of apoptotic mechanisms. Among flavanone derivatives, HST and HSD have demonstrated significant antiproliferative properties across a spectrum of human cancer cell lines. While it is imperative to exercise caution when interpreting findings derived from diverse cellular culture environments, the most pronounced antiproliferative responses elicited by HST and HSD, characterized by lower corresponding half-maximal inhibitory concentration (IC 50) concentrations, have been documented for breast cancer cell lines including MCF-7 cells exhibiting resistance to MDA-MB-231 and doxorubicin (16–18). Moreover, HSD has been identified as a highly effective antiproliferative mediator in several cancer cell lines, including those derived from leukemia (NALM6 and K-562) and lymphoma (Ramos), among others (19, 20). Furthermore, HST has been noted to exhibit superior antiproliferative properties against cell lines originating from cervical (HeLa), CRC (HT-29), and skin or ocular (A-431) malignancies (21–23). Additionally, in numerous cancer cell lines, no significant reduction in proliferation was observed, indicating the necessity for further investigation into cell line-specific toxicity and clinical implications (24, 25). Concerning the structure-activity relationship, limited studies have conducted comparative analyses of the antiproliferative impacts of HST and HSD, as well as their structural analogs. In HL-60 leukemia cells, a marked reduction in cell viability was observed when these cells were subjected to HST, in contrast to HSD (26), indicating that this particular cell line exhibited heightened susceptibility to apoptosis triggered by HST, thereby implying that the presence of the rutinoside moiety in HSD mitigated its apoptotic impacts within this specific cell line. Despite encouraging findings, most studies on the antiproliferative activity of HSD and HST have been performed *in vitro*, often using high concentrations that may not be physiologically achievable in humans. Furthermore, the heterogeneity among CRC cell lines and experimental conditions makes it difficult to draw firm conclusions about clinical relevance. The limited number of *in vivo* studies underscores the need for more rigorous animal and translational research to confirm these antiproliferative properties under conditions that better reflect human CRC.

## Chemopreventive impacts of HSD and HST

Hesperetin and HSD have garnered considerable attention for their chemopreventive potential in CRC. Chemoprevention contains the use of natural or synthetic mediators to prevent, delay, or reverse carcinogenesis, and both HSD and HST have demonstrated efficacy in all 3 phases of cancer development: initiation, promotion, and progression. Early studies laid the groundwork for understanding HSD’s preventive potential in chemically induced CRC models (27). More recent work has expanded on these findings. For instance, Li et al. (28) demonstrated that HSD impairs colon cancer cell growth and migration by targeting SLC5A1-EGFR signaling (28). Similarly, another 2024 study showed that HSD suppresses glycolysis via downregulation of HK2, GLUTs, and PFKFBs in both cell lines and xenografts (29). Additionally, a 2023 report found a synergistic chemopreventive effect when HSD is combined with gallic acid, leading to enhanced inhibition of CRC spheroid formation and stemness (30).

In experimental models of chemically induced colorectal carcinogenesis, HSD significantly suppressed the creation of aberrant crypt foci (ACF), a preneoplastic marker in CRC. HSD has also been shown to exhibit chemopreventive effects by modulating phase I and phase II detoxifying enzymes. It induces quinone reductase and glutathione S-transferase (GST), enzymes that play important functions in the detoxification of carcinogens, and suppresses cytochrome P450 isoenzymes involved in procarcinogen activation (31). These effects collectively contribute to reduced DNA adduct formation and oxidative impairment, which are markers of cancer initiation. In addition to antioxidant effects, HSD and HST exhibit anti-inflammatory properties that support their chemopreventive role. Chronic inflammation is a known contributor to CRC development, and both compounds downregulate key inflammatory mediators like TNF-α, NF-κB, IL-6, COX-2, and iNOS. For example, in a study where 1,2-dimethylhydrazine (DMH) was administered to make colon inflammation and oxidative stress, HST treatment significantly reduced the expression of these pro-inflammatory proteins and improved histopathological markers like goblet cell integrity and mucin preservation (32). Furthermore, HST has been observed to modulate cell cycle arrest and apoptosis, both of which are crucial for eliminating damaged or pre-cancerous cells. It upregulates pro-apoptotic proteins (caspase-9, Bax, caspase-3) and downregulates anti-apoptotic Bcl-2, thereby promoting programmed cell death in transformed colonic epithelial cells (33). Collectively, these findings suggest that HSD and HST exert their chemopreventive effects through multiple mechanisms, including antioxidant defense enhancement, anti-inflammatory activity, induction of apoptosis, and modulation of detoxifying enzymes. Their favorable safety profile and dietary origin further strengthen their candidacy as chemopreventive agents for CRC. Although multiple animal studies suggest chemopreventive benefits of HSD and HST, these models are often based on chemically induced carcinogenesis and do not fully capture the complex, multifactorial nature of human CRC. Differences in dosing schedules, duration of treatment, and dietary context further complicate comparisons between studies. In addition, the long-term safety and effective human dosage of these flavonoids remain undefined, highlighting the need for standardized protocols and clinical investigations before their chemopreventive role can be established.

## Anti-inflammatory effects of HST and HSD

Numerous *in vivo* and *in vitro* investigations have been undertaken to evaluate the efficacy of HSD and HST, along with its metabolites and synthetic derivatives, in mitigating the previously mentioned inflammatory responses (34, 35). Variations in the experimental paradigms employed can significantly influence the results of these studies, necessitating distinct interpretations. For instance, it has been observed that in the ovalbumin (OVA)-induced inflammation model, the concentrations of cytokines, like IL-4, in bronchoalveolar lavage fluid escalate considerably higher than in models of inflammation induced by lipopolysaccharide (LPS) (36, 37). Consequently, the immunomodulatory properties of HSD may manifest as immunosuppression within the OVA model, whereas they may exhibit immunopotentiation in the context of the LPS model (38). Furthermore, the observation that the predominant fraction of bioavailable flavonoid occurs in a metabolized form complicates the extrapolation of *in vitro* findings to *in vivo* scenarios (39). The anti-inflammatory potential of HSD and HST has been supported by both *in vitro* and *in vivo* studies, particularly through suppression of NF-κB and related cytokines. However, many of these experiments rely on acute or induced inflammation models that may not reflect the chronic inflammatory environment associated with CRC progression. Moreover, bioactive metabolites rather than the parent compounds are more likely to circulate systemically, and their contributions to anti-inflammatory effects are not yet fully understood. These limitations highlight the need for further mechanistic studies and clinical validation.

## Antioxidant impacts of HST and HSD

The antioxidant activities of HSD and HST have been previously documented (7). In contemporary research, considerable emphasis has been directed toward elucidating the protecting attributes of HST and HSD in relation to numerous oxidants, including hydrogen peroxide and peroxynitrite, as well as a multitude of other toxins and chemicals that inflict damage upon tissues through mechanisms associated with oxidative stress or alternative pathways (40, 41). HST and HSD manifest their antioxidant capabilities through two primary mechanisms: direct scavenging of radicals and the improvement of cellular antioxidant defenses. A plethora of investigations have demonstrated that HSD effectively neutralizes reactive oxygen species (ROS), which encompass hydroxyl radicals, superoxide anions, nitric oxide radicals, and peroxynitrite (42, 43). This direct radical scavenging role of HST and HSD is pivotal in safeguarding DNA, proteins, and tissues from damage instigated by both extrinsic and intrinsic factors. According to extant research, HSD treatment led to a decrease in the concentrations of all biomarkers, and the antioxidant status was restored to near-normal levels. Consequently, HSD provides a protective impact against tissue damage induced by various intrinsic and extrinsic factors (44, 45). Furthermore, numerous studies have demonstrated that HSD mitigates cellular damage by enhancing cellular defenses. HSD facilitated or upregulated the expression of genes associated with ERK 1/2 and Nrf2 (46, 47). The stimulation of ERK 1/2 and Nrf2 engendered the upregulation of HO-1 expression, culminating in a decrease of intracellular pro-oxidants and an enhance in bilirubin, which acts as an endogenous antioxidant. Additionally, HO-1 expression augmented the levels of carbon monoxide (CO), which possesses anti-inflammatory and anti-apoptotic properties in cellular contexts. Also, CO enhanced the activity of guanylyl cyclase within cells (11). Moreover, Nrf2 further elevated antioxidant enzymes levels, including superoxide dismutase (SOD) and catalase (CAT). Thus, HSD enhanced the cellular antioxidant defense capacity via the induction of HO-1 through the ERK/Nrf2 signaling pathway. The antioxidant activities of HSD and HST are well established in preclinical settings, both through direct radical scavenging and via modulation of Nrf2-related signaling pathways. Nevertheless, most evidence comes from rodent or cell culture studies, often using concentrations higher than those attainable through dietary intake. Furthermore, their poor bioavailability and rapid metabolism pose challenges for translation to humans. These issues suggest that while antioxidant effects are promising, improved delivery systems and clinical trials are necessary to confirm their significance in CRC prevention and therapy.

## The effects of HSD on antiproliferative activity, chemopreventive, anti-inflammatory, and antioxidant effects in colon cancer

**FIGURE 1:**
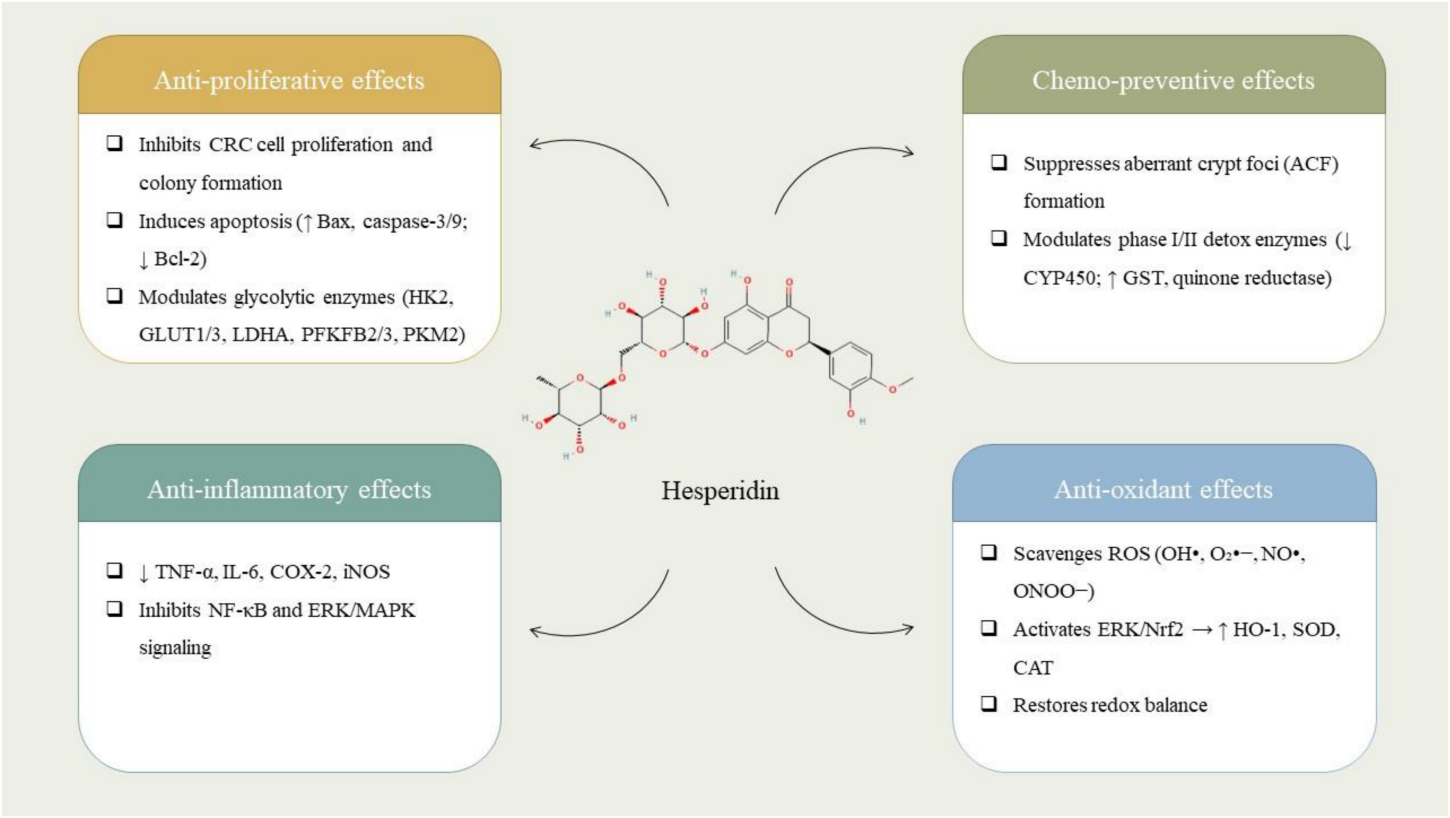
Chemical structure of hesperidin (HSD) and its reported biological effects in colorectal cancer. HSD exerts antiproliferative activity by inhibiting cell viability, colony formation, and glycolytic enzyme expression; displays chemopreventive properties by reducing aberrant crypt foci and modulating phase I/II detoxifying enzymes; shows anti-inflammatory effects through downregulation of NF-κB, COX-2, iNOS, and pro-inflammatory cytokines; and enhances antioxidant defense by scavenging ROS and activating the ERK/Nrf2/HO-1 signaling pathway.

Hesperidin compound exhibits multiple bioactivities relevant to cancer prevention and treatment, including the ability to inhibit abnormal cell proliferation, prevent tumor development, suppress inflammation, and counteract oxidative stress (Figure 1). These effects are interconnected and play crucial roles in the beginning and development of cancer and other chronic diseases. The title reflects a comprehensive approach aligned with predictive, preventive, and personalized medicine, emphasizing HSD’s relevance in modern health strategies. Its natural origin and multi-targeted actions make it an attractive candidate for further research and clinical applications in both oncology and chronic disease management. A comprehensive investigation was conducted to elucidate the effects of HSD on glycolytic processes and tumor progression within CRC cell lines, specifically HCT116 and SW620. *In vitro* experimental assays demonstrated that HSD, administered at levels ranging from 0 to 500 μmol/L, significantly suppressed the migration, proliferation, and colony formation of CRC cells. Furthermore, HSD was found to induce apoptotic processes and modify the cell cycle progression. At a mechanistic level, HSD was observed to downregulate crucial glycolytic enzymes and transporters, which include hexokinase 2, GLUT3, GLUT1, LDHA, PFKFB3, PFKFB2, and PKM2. *In vivo* studies revealed that HSD treatment effectively inhibited tumor growth in nude mice models. Pathway analyses elucidated that HSD significantly influenced metabolic functions, underscoring its potential as an anti-cancer agent targeting the metabolic pathways associated with CRC (29). A comprehensive investigation was conducted to elucidate the impacts of HSD on human CRC cells (HCT116) and its interactions with human hemoglobin (HHb). HSD instigated membrane impairment, suppressed colony formation, and initiated oxidative stress alongside mitochondrial dysfunction in CRC cells. It facilitated apoptosis via the upregulation of caspase-9, caspase-3, and Bax/Bcl-2, while also inducing the release of cytochrome c. Furthermore, HSD established a stable complex with HHb via hydrogen bonding, which resulted in partial protein unfolding, as corroborated by spectroscopy, molecular docking, and dynamic simulations (48).

Colorectal cancer is a widespread malignancy that frequently manifests with non-specific symptoms, whereas traditional chemotherapy regimens are associated with notable adverse effects. HSD, recognized for its antioxidant properties, has the potential to mitigate these detrimental effects; however, its therapeutic efficacy is constrained by its inadequate bioavailability. A recent investigation sought to address this limitation by encapsulating HSD within poly (lactic-co-glycolic acid) nanoparticles and evaluating their impact on HCT116 CRC cell lines. The nanoparticles exhibited a remarkable drug loading efficiency of 90%, demonstrated a uniform spherical morphology with an average diameter of 76.2 nm, and facilitated a sustained drug release profile of 93% over a duration of 144 h. Following a 48-h exposure, the nanoparticles significantly impeded the viability of cancer cells, particularly at a value of 10 μg/mL (14). The preservation of colon homeostasis is imperative for the mitigation of colon cancer, with a well-balanced gut microbiota serving a pivotal function in this regulatory mechanism. Probiotics, exemplified by *Lacticaseibacillus rhamnosus* GG (LGG), have the capacity to modulate gut microbial diversity and generate metabolites that foster a salutary colonic milieu. The oncofetal gene CYP2W1, which is typically silenced postnatally, exhibits re-expression in colorectal malignancies and correlates with the severity of the disease. A research investigation assessed the impact of HSD-treated LGG cell-free supernatants (CFS) on CaCo2 colon cancer cell lines. The findings indicated that diluted HSD-treated CFS markedly reduced the viability of cancer cells and inhibited CYP2W1 gene expression, nearly achieving complete silencing. This observation implies a prospective anticancer effect of HSD-fortified probiotic metabolites (49).

A research investigation examined the synergistic anticancer properties of gallic acid and HSD in the context of CRC. Both compounds, sourced from Hakka pomelo tea (HPT) and extracted utilizing ethyl acetate, demonstrated a significant inhibition of CRC cell proliferation, particularly within the HCT-116 and HT-29 cell lines. The amalgamated extract exhibited superior inhibitory effects on cell viability in comparison to either compound administered individually. From a mechanistic perspective, the combination elicited G1-phase cell cycle arrest and upregulated Cip1/p21, resulting in diminished stemness (CD-133), proliferation (Ki-67), and spheroid formation in a three-dimensional culture model, thereby indicating the potential for enhanced CRC therapeutic strategies through this natural combination approach (30). The research examined the chemopreventive properties of HSD within a DMH-induced CRC rat model, emphasizing its influence on the modulation of activin A and Smad4 signaling pathways. Exposure to DMH resulted in an upregulation of activin A expression, an increase in oxidative stress markers (MDA, NO), and a downregulation of Smad4 expression accompanied by diminished levels of antioxidant enzymes (GSH, SOD). The administration of HSD, either concurrently or post-DMH exposure, significantly enhanced the expression of Smad4 and activin A while restoring antioxidant levels, thereby suggesting that HSD has the potential to attenuate CRC progression by bolstering antioxidant defenses and modulating tumor-suppressive signaling pathways (15).

In a research investigation, HSD-incorporated Zn^2+^@SA/PCT nanocomposites were synthesized and systematically assessed for their anticancer efficacy against HCT116 colon carcinoma cells. The findings demonstrated that the nanocomposites significantly impeded cellular proliferation and triggered apoptosis-mediated necrosis. This phenomenon was facilitated through the upregulation of apoptotic proteins, thereby substantiating the potential of the HSD nanocomposite as a promising therapeutic modality for the treatment of colon cancer (50). Another investigation elucidated the impacts of HSD on the Aurora-A protein and the PI3K/Akt/mTOR signaling cascade within a mouse model of colon carcinoma induced by azoxymethane (AOM). Treatment with HSD (both at the initiation stage and after initiation) substantially facilitated apoptosis via the control of the Bax/Bcl-2 ratio, an elevation in cytochrome c release, and the stimulation of caspases 3 and 9. Furthermore, it augmented the p53-p21 tumor suppressor pathway, elevated the PTEN expression, and diminished the levels of phosphorylated Akt, PI3K, and mTOR, consequently promoting autophagy via LC3-II and Beclin-1. In addition, HSD exerted inhibitory effects on Aurora-A, a critical upstream activator of the PI3K/Akt pathway, and reinstated GSK-3β activity, thereby averting the accumulation of oncoproteins such as c-Jun, β-catenin, and c-Myc. These results substantiate the potent anticancer efficacy of HSD through the modulation of apoptotic, autophagic, and oncogenic pathways (51).

An investigation assessed the chemopreventive properties of HSD utilizing an AOM-induced murine model of colon carcinoma. HSD was administered during either the initiation phase or the post-initiation phase of tumor development. The data indicated that HSD significantly attenuated the formation of ACF, reduced tumor incidence, and mitigated oxidative stress, while simultaneously increasing antioxidant levels. Moreover, HSD treatment caused a decrease in the proliferation biomarker PCNA, and effectively inhibited inflammatory responses by downregulating NF-κB, iNOS, and COX-2 expression (52). An investigation analyzed the chemoprotective properties of Satsuma mandarin juice and its fortified variants (MJ5 and MJ2), which are characterized by elevated concentrations of beta-cryptoxanthin and HSD, in the context of AOM-induced colorectal carcinogenesis in rodent models. Following AOM administration, the subjects were administered MJ, MJ2, or MJ5 via their drinking water over a duration of 36 weeks. All experimental treatments markedly diminished both the incidence and multiplicity of colonic adenocarcinomas, attenuated cellular proliferation markers (PCNA, cyclin D1), and augmented apoptotic activity within neoplastic tissues. The results of investigation exhibited that the consumption of mandarin juice, which is abundant in beta-cryptoxanthin and HSD, may confer protective effects against colon cancer by impeding tumorigenesis and facilitating programmed cell death (53).

Another work explored the impacts of diosmin and HSD, both separately and in conjunction, on AOM-induced colorectal carcinoma in male F344 rat specimens. Interventions were administered during either the post-initiation or initiation stages of carcinogenesis. Both flavonoids, whether utilized in isolation or in combination, demonstrated a substantial reduction in both the multiplicity and incidence of colonic neoplasms when contrasted with AOM alone. Nevertheless, the synergistic application did not exhibit any additional advantages over the individual treatments. Furthermore, all interventions markedly suppressed the development of ACF and diminished biomarkers related to cellular proliferation, such as the BrdU labeling index, nuclear organizer regions, ornithine decarboxylase activity, and polyamine concentrations. These results imply that diosmin and HSD possess chemopreventive properties against the process of colon carcinogenesis (27). Oxaliplatin (OXP) represents a prevalent therapeutic intervention for CRC; however, the concomitant administration of pharmacological agents may mitigate resistance and adverse effects. The investigation devised nanoliposomes that simultaneously deliver HSP and OXP, encapsulated within cationic Okra gum to enhance stability. The formulated coated liposomes exhibited a controlled, pH-sensitive release profile of HSP and OXP, while maintaining stability for an approximate duration of 30 days. The combination liposomes displayed a markedly elevated cytotoxic effect against HT-29 colon cancer cells after a 48-h exposure when compared to each drug administered independently, indicating a potential augmentation in therapeutic efficacy (54). Overall, it was documented that HSD has significant antiproliferative, anti-inflammatory, chemopreventive, and antioxidant impacts, positioning it as a promising bioactive compound in the management and prevention of cancer and other inflammation-associated diseases. Its ability to inhibit abnormal cell growth, modulate key molecular pathways involved in apoptosis and autophagy, and suppress oxidative stress and inflammatory responses underscores its therapeutic potential. Evidence from both *in vivo* and *in vitro* studies supports HSD’s role in reducing tumor incidence, downregulating pro-inflammatory mediators, and enhancing cellular antioxidant defenses.

## The effects of HST on antiproliferative activity, anti-inflammatory, chemopreventive, and antioxidant effects in colon cancer

**FIGURE 2:**
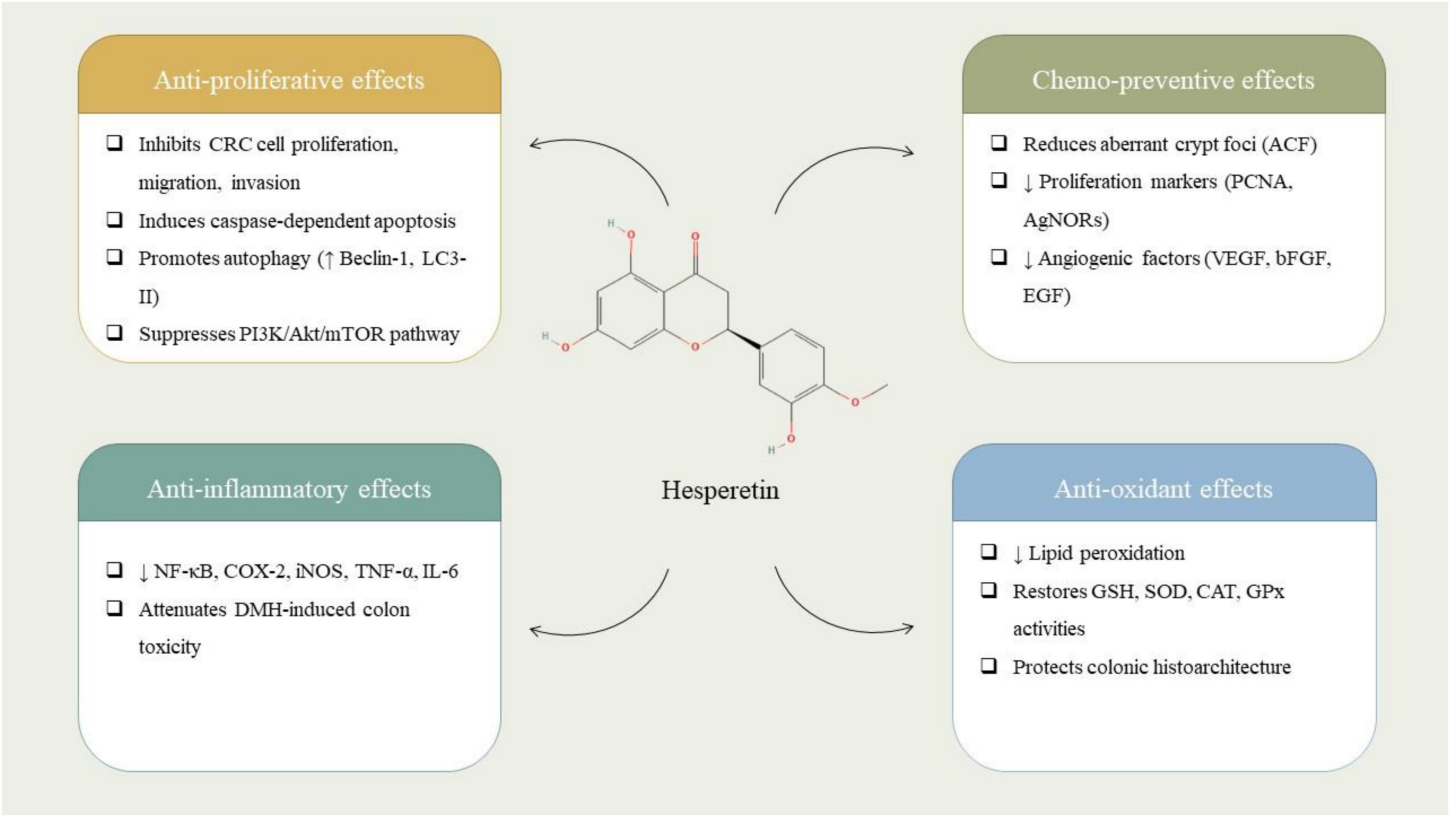
Chemical structure of hesperetin (HST) and its documented effects in colorectal cancer. HST reduces tumor cell proliferation and migration, induces caspase-dependent apoptosis and autophagy, and suppresses PI3K/Akt/mTOR signaling. It demonstrates chemopreventive potential by decreasing aberrant crypt foci, proliferation markers, and angiogenic factors, while exerting anti-inflammatory actions via inhibition of NF-κB, COX-2, iNOS, and pro-inflammatory cytokines. HST also exhibits antioxidant activity by restoring GSH, SOD, CAT, and GPx levels and reducing lipid peroxidation in experimental CRC models.

The investigation of the antiproliferative, anti-inflammatory, chemopreventive, and antioxidant impacts of HST is of growing importance in the field of biomedical and cancer research. HST has involved considerable attention due to its broad-spectrum biological activities and low toxicity profile. These properties make it a compelling candidate for integrative therapeutic strategies aimed at slowing or preventing the progression of several chronic diseases, including cancer. Understanding how HST exerts its effects at the molecular and cellular levels, by modulating key signaling pathways, reducing oxidative damage, suppressing chronic inflammation, and inhibiting uncontrolled cell proliferation, can contribute to the development of more effective and personalized interventions (Figure 2). Moreover, as natural compounds gain prominence in predictive and preventive medicine, elucidating the multifaceted actions of HST may open new avenues for the use of dietary flavonoids in clinical and public health settings. A research investigation has devised an innovative targeted drug delivery system employing DCLK1-functionalized, folic acid-conjugated HST encapsulated within chitosan nanoparticles (CFH-DCLK1) to preferentially target cancer stem cells (CSCs) within the context of CRC. Within HCT116 colon cancer cellular models, CFH-DCLK1 nanoparticles substantially promoted apoptotic processes while concurrently inhibiting cellular migration and invasion. In comparison to non-targeted CFH nanoparticles, the administration of CFH-DCLK1 resulted in a more pronounced downregulation of CSC-associated markers, which include DCLK1, STAT1, and NOTCH1. Moreover, within a three-dimensional spheroid model, these nanoparticles significantly impeded colonosphere formation, thereby underscoring their potential as a viable therapeutic strategy for CSC-targeted interventions in CRC (55).

A research investigation examined the anti-neoplastic efficacy of a HST-graphene oxide nanocomposite (HST-GO) against LS174t colon carcinoma cells. Investigators assessed the impacts of HST in isolation, graphene oxide nanosheets (GONS), and HST-encapsulated GONS. The findings indicated that HST-GO exhibited the most pronounced cytotoxic and pro-apoptotic properties, resulting in a substantial reduction in cell viability. Molecular assessments demonstrated a downregulation of the anti-apoptotic protein BCL2 and an upregulation of the pro-apoptotic protein BAX, thereby validating the induction of apoptosis. These results indicated that HST-GO represents a promising nanotherapeutic candidate for the management of colon cancer (56). Another research investigation examined the underlying mechanisms via which HST employs its anticancer effects on HCT-15 colon cancer cell lines. Administration of HST (at concentrations of 25 and 50 μM) significantly diminished cell proliferation and viability in a manner that was both dose-dependent and time-dependent, primarily through the induction of caspase-dependent apoptosis. Furthermore, HST was observed to inhibit the PI3K/Akt signaling pathway by attenuating Akt phosphorylation and upregulating PTEN expression. In addition, it facilitated autophagy by dephosphorylating mTOR and enhancing the levels of Beclin-1 and LC3-II. Importantly, HST was found to augment the anticancer effects of Akt and mTOR inhibitors (specifically rapamycin and LY294002), thereby suggesting a synergistic potential in the therapeutic targeting of colon cancer (57).

A research investigation scrutinized the chemopreventive properties of HST concerning DMH-induced colorectal malignancy in male Wistar rat specimens. HST was administered at various stages: during the initiation phase, post-initiation phase, or continuously over the full duration of the 32-week study period. The intake of HST caused a significant attenuation of cellular proliferation indicators (AgNORs, PCNA), an enhancement of apoptotic processes, and a reduction in the concentrations of angiogenic growth factors (bFGF, VEGF, EGF). Furthermore, COX-2 mRNA expression was significantly diminished in both mucosal and fecal specimens. These findings implied that HST serves a protective role in the context of colon carcinogenesis by obstructing cellular proliferation, inflammatory responses, and angiogenesis while simultaneously facilitating apoptotic mechanisms (58). Another study evaluated the apoptosis-inducing properties of HST and its derivative (HA) on HT-29 colon carcinoma cells. Both substances exhibited cytotoxic effects that were dependent on the duration and dosage of exposure, with a 50% inhibition of cellular growth observed at 70 μM HST and 32 μM (HA) following a 24-h incubation period. The presence of apoptotic characteristics was validated through AO/EB staining, the loss of mitochondrial membrane potential, and the assessment of DNA damage utilizing the comet assay. Additionally, the treated cells exhibited heightened oxidative stress, as evidenced by increased levels of TBARS and PCC, alongside a modest decrease in the activities of antioxidant enzymes (CAT, SOD, GPx). Western blot analyses demonstrated an upregulation of pro-apoptotic proteins (cleaved caspase-3, Bax, cytochrome c) and a downregulation of the anti-apoptotic protein Bcl-2, thereby corroborating the induction of apoptosis by HST and HA (59).

A work assessed the impact of HST on lipid peroxidation, the antioxidant defense mechanism, and the histoarchitecture of the colon in male Wistar rats subjected to DMH-induced colon carcinogenesis. The intake of DMH resulted in an elevation of lipid peroxidation levels, alongside a decrease in the activities of antioxidant enzymes (GR, SOD, CAT, GPx) and a decrease in GSH concentrations. Supplementation with HST, irrespective of whether it was applied during the initiation phase, after the initiation phase, or consistently throughout the carcinogenic process, effectively ameliorated these alterations, suggesting a protective role against oxidative stress and colonic injury (60). An investigation assessed the protective effects of HST on the formation of ACF and the activity of xenobiotic-metabolizing enzymes during DMH-induced colon carcinogenesis in male Wistar rats. Exposure to DMH resulted in a heightened occurrence of ACF, an elevation in phase I enzyme activities, and a reduction in phase II enzyme activities within both the hepatic and colonic mucosal tissues. HST was administered at various stages: post-initiation, initiation, and continuously throughout the entire duration of 32 weeks. The supplementation of HST significantly mitigated ACF formation, restored enzyme activities to normative levels, and these chemopreventive effects were most pronounced when HST was administered throughout the entire experimental timeline (61). Another study evaluated the effects of varying doses of HST on the enzymatic activity of bacteria and the formation of ACF in DMH-induced colon cancer models in male Wistar rats. DMH administration resulted in an augmentation of the activity of several fecal and colonic mucosal bacterial enzymes, concomitantly leading to a significant increase in ACF formation. The supplementation of HST, especially at a dosage of 20 mg/kg, markedly diminished bacterial enzyme activity and inhibited the progression of ACF. These results indicate that HST possesses considerable chemopreventive potential in the context of colon carcinogenesis (62).

The aim of a work was the evaluation of HST intake on the prevalence of ACF, the extent of lipid peroxidation, and the status of antioxidant defenses in male Wistar rats subjected to DMH-induced colon carcinogenesis. The administration of DMH was seen to elevate both tumor incidence and the quantity of ACF, concurrently diminishing the activities of key antioxidant enzymes (GPx, GST, CAT, SOD) and promoting lipid peroxidation. The administration of HST at various dosages markedly attenuated tumor incidence and ACF, while also reinstating the activities of antioxidant enzymes and normalizing lipid peroxidation levels, thereby underscoring its protective and chemopreventive properties (63). Another investigation assessed the protective properties of HST in mitigating colon toxicity provoked by the carcinogenic agent DMH in Wistar rats. Administration of a singular dose of DMH resulted in the generation of excessive ROS, elevated lipid peroxidation levels, diminished antioxidant defenses, and exacerbated inflammatory responses within the colon. Prolonged oral administration of HST over a period of 14 days markedly diminished lipid peroxidation, enhanced the activities of various antioxidant enzymes (GST, GSH, GR, GPx, SOD), and reduced levels of inflammatory proteins (i-NOS, IL-6, COX-2, TNF-α, NF-kB-p65). Furthermore, HST exhibited protective effects against the damage inflicted upon goblet cells and the subsequent loss of mucin attributable to DMH, thereby underscoring its role as a protective agent against colon toxicity (32). To facilitate comparison, Table 1 summarizes the most relevant studies investigating HSD in CRC models, while Table 2 provides an overview of HST studies.

**TABLE 1:** The effects of hesperidin on antiproliferative activity, chemopreventive effects and molecular signaling in colorectal cancer.

Dosage of hesperidin	Duration of study	Model	Main effects	References
0–500 μM (CCK-8 and colony assays); 0–75 μM (migration assays)	Not specified (acute cell treatment)	*In vitro* (HCT116, SW620)	Inhibited viability and colony formation; suppressed migration; induced apoptosis and cell cycle arrest; downregulated glycolysis-related proteins (HK2, GLUT1/3, LDHA, PFKFB2/3, PKM2)	(29)
Not specified (oral or injection)	4 weeks	*In vivo* (nude mice xenograft)	Reduced tumor volume and weight; no effect on body weight; suppressed glycolysis-related genes; altered metabolic pathways (GO/KEGG)	(29)
10–500 μM	24–72 h	*In vitro* (HCT116)	Induced oxidative stress and mitochondrial dysfunction; membrane damage; inhibited colony formation; upregulated Bax, caspase-9/3; downregulated Bcl-2; cytochrome c release	(48)
Molecular-level assays	N/A	*In vitro* (human hemoglobin binding)	Formed static complex with hemoglobin via H-bonds; partial unfolding of protein; confirmed by spectroscopy, docking, and dynamics simulations	(48)
PLGA-loaded HSD (10 μg/mL, lowest effective)	48 h (MTT); release up to 144 h	*In vitro* (HCT116)	∼90% encapsulation efficiency; spherical nanoparticles (∼76 nm); sustained release (∼93% at 144 h); significantly reduced cell viability; improved delivery vs. free HSD	(14)
HSD-treated LGG culture (CFS tested on CaCo2)	24–48 h	*In vitro* (CaCo2)	Reduced CaCo2 viability; strong downregulation of CYP2W1 gene; showed probiotic–HSD synergy	(49)
Part of Hakka pomelo tea extract	24–72 h	*In vitro* (HT-29, HCT-116, 3D spheroids)	Higher inhibition than HSD or gallic acid alone; G1 arrest; upregulated Cip1/p21; downregulated Ki-67 and CD133; suppressed spheroid formation	(30)
125 μM HSD + 1.125 μM OXP (nanoliposomes)	48 h	*In vitro* (HT-29)	Co-delivery enhanced cytotoxicity; drug release (HSD 98%, OXP 66% in 24 h); improved stability with COG coating; CAT identified as common target	(54)
∼100 mg/kg (typical)	10–16 weeks	*In vivo* (DMH-induced CRC, rat)	Upregulated Smad4 and activin A; decreased MDA and NO; increased GSH and SOD; restored antioxidant balance; chemopreventive via Smad4/Activin A signaling	(15)
Zn^2+^@SA/PCT-HSD nanocomposite	24–72 h	*In vitro* (HCT116)	Inhibited growth; induced apoptosis and necrosis; upregulated apoptotic proteins; demonstrated nanocomposite anticancer potential	(50)
Not precisely specified	Initiation and post-initiation	*In vivo* (AOM-induced CRC, mouse)	Inhibited Aurora-A kinase; downregulated PI3K/Akt/mTOR; upregulated PTEN and p53-p21; induced apoptosis (Bax/Bcl-2, caspase-3/9, cyt c release); promoted autophagy (Beclin-1, LC3-II); suppressed β-catenin, c-Myc, c-Jun	(51)
Not specified (dietary, mg/kg)	After AOM injection (initiation and post-initiation)	*In vivo* (Swiss albino mouse)	Reduced aberrant crypt foci and tumor incidence; decreased PCNA index; reduced oxidative stress; increased antioxidants; suppressed NF-κB, iNOS, COX-2; showed anti-inflammatory and antiproliferative effects	(52)
Part of citrus peel extracts (MJ2 and MJ5 rich in HSD)	36 weeks (after AOM)	*In vivo* (F344 rats with AOM-induced CRC)	Lowered adenocarcinoma incidence/multiplicity; decreased PCNA and cyclin D1; increased apoptotic index; mechanism: suppressed proliferation, induced detox enzymes	(53)
1000 ppm HSD alone or 100 ppm HSD + 900 ppm diosmin (diet)	32 weeks (initiation + post-initiation)	*In vivo* (F344 rats with AOM-induced CRC)	Decreased colon neoplasm incidence/multiplicity; reduced aberrant crypt foci; lowered BrdU labeling, AgNORs, ODC activity, and blood polyamines; no synergy with diosmin	(27)

**TABLE 2:** The effects of hesperetin on antiproliferative activity, chemopreventive effects and molecular signaling in colorectal cancer.

Dosage of hesperetin	Duration of study	Model	Main effects	References
25–100 μM	24–72 h	*In vitro* (SW480 cells)	Reduced proliferation and migration; induced apoptosis through caspase activation; caused cell cycle arrest	(56)
∼100 mg/kg (oral)	10–16 weeks	*In vivo* (DMH-induced CRC, rat)	Lowered aberrant crypt foci; reduced PCNA and VEGF expression; restored antioxidant enzymes (SOD, CAT, GPx)	(57)
20–80 μM	24–72 h	*In vitro* (HT-29 cells)	Reduced oxidative stress; decreased lipid peroxidation; restored GSH and CAT activity; induced apoptosis	(58)
50 mg/kg (oral)	Xenograft duration (4–6 weeks)	*In vivo* (HCT-116 xenograft, nude mice)	Suppressed tumor growth; downregulated PI3K/Akt/mTOR pathway; promoted autophagy (↑ Beclin-1, LC3-II)	(59)
∼50–100 mg/kg (dietary)	Initiation and post-initiation phases	*In vivo* (DMH-induced CRC, rat)	Inhibited NF-κB activation; reduced COX-2, iNOS, TNF-α, IL-6; exhibited anti-inflammatory and antiproliferative effects	(60)
10–50 μM	24–48 h	*In vitro* (LoVo cells)	Induced apoptosis; downregulated Bcl-2; upregulated Bax and caspase-3/9; caused mitochondrial dysfunction	(61)
50 mg/kg + 5-FU (co-treatment)	4 weeks	*In vivo* (CRC xenograft mouse)	Enhanced 5-FU cytotoxicity; reduced tumor volume more effectively than single agents; suggested chemosensitizing effect	(62)
25–100 μM	24–72 h	*In vitro* (SW480 cells)	Reduced proliferation and migration; induced apoptosis through caspase activation; caused cell cycle arrest	(63)
∼100 mg/kg (oral)	10–16 weeks	*In vivo* (DMH-induced CRC, rat)	Lowered aberrant crypt foci; reduced PCNA and VEGF expression; restored antioxidant enzymes (SOD, CAT, GPx)	(32)
20–80 μM	24–72 h	*In vitro* (HT-29 cells)	Reduced oxidative stress; decreased lipid peroxidation; restored GSH and CAT activity; induced apoptosis	(55)

## Future research directions and clinical implications

Future research should focus on bridging the gap between clinical applications and preclinical findings of HST and HSD in CRC. Although numerous *in vivo* and *in vitro* studies have demonstrated promising antiproliferative, chemopreventive, antioxidant and anti-inflammatory impacts, clinical trials evaluating the safety, efficacy, pharmacodynamics, and pharmacokinetics of these flavonoids in humans remain limited. To date, only a few clinical trials have investigated HSD or HST in humans, and most of these focus on cardiovascular or metabolic endpoints rather than cancer (64). No large-scale clinical trials specifically targeting CRC prevention or therapy with these compounds have been reported, underscoring a critical research gap. Nevertheless, preliminary data from non-cancer trials indicate favorable safety profiles, which provide a rationale for future CRC-focused investigations.

Well-designed, randomized, placebo-controlled trials are essential to confirm the beneficial possible of HST and HSD in different stages of CRC, from prevention in high-risk populations to adjunctive treatment in patients undergoing chemotherapy or radiotherapy. Moreover, future investigations should aim to elucidate the precise molecular targets and signaling pathways modulated by these compounds in various tumor microenvironments, including their effects on the gut microbiota, immune response, and cancer stem cells. Advanced omics technologies like proteomics, transcriptomics, and metabolomics could offer deeper insight into the global regulatory networks influenced by HSD and HST. Given the poor water solubility and limited bioavailability of these flavonoids, future research should also prioritize the development of new delivery systems, like liposomes, nanoparticles, or polymer-based carriers, to enhance their stability, targeted delivery, and therapeutic index. Another important direction involves the investigation of synergistic effects of HSD and HST with conventional chemotherapeutic agents, such as oxaliplatin or 5-fluorouracil, as well as with other natural compounds. Understanding the interactions between these agents may reduce toxicity, overcome drug resistance, and improve overall treatment outcomes. Additionally, personalized medicine approaches, including the identification of patient-specific biomarkers and genetic polymorphisms, may help determine which individuals are most likely to benefit from flavonoid-based interventions. While HSD and HST are generally regarded as safe due to their dietary origin, high-dose supplementation has occasionally been associated with gastrointestinal discomfort, headaches, or allergic reactions in sensitive individuals. Moreover, their potential interactions with conventional chemotherapeutic drugs or other medications have not been systematically studied. These aspects highlight the importance of careful safety monitoring in future clinical trials.

Finally, translational research should evaluate the long-term effects and potential adverse reactions of HSD and HST supplementation, especially in populations with co-morbid conditions or those taking multiple medications. The integration of these compounds into dietary guidelines, functional foods, or nutraceuticals for CRC prevention could have significant public health implications if supported by robust scientific evidence. Together, these points suggest that while HSD and HST hold promise as adjuncts in CRC prevention and therapy, rigorous clinical validation remains the critical next step.

## Conclusion

Hesperetin and HSD are two citrus-derived flavonoids, which have emerged as promising bioactive compounds with significant therapeutic potential against CRC. Accumulating evidence from *in vivo* and *in vitro* studies highlights their multifaceted impacts, including chemopreventive, anti-inflammatory, antiproliferative, and antioxidant properties. These impacts are facilitated via the control of important molecular pathways, and apoptotic signaling cascades. Both compounds have shown the capability to suppress progression, tumor initiation, and metastasis, as well as to enhance the effectiveness of conventional chemotherapeutic mediators while mitigating their associated toxicities. Importantly, HSD and HST exhibit potential in regulating oxidative stress by enhancing the endogenous antioxidant defense systems, like GPX, CAT, and SOD, thereby protecting normal tissues from carcinogen-induced damage. Additionally, they modulate inflammatory responses by downregulating enzymes and pro-inflammatory cytokines such as COX-2, IL-6, TNF-α, and iNOS. These actions contribute to maintaining redox homeostasis and reducing the chronic inflammation that underlies colorectal tumorigenesis. Despite these encouraging findings, most studies to date have been preclinical, and translation to clinical settings remains limited. There is a need for further investigation into their pharmacokinetics, optimal dosing strategies, long-term safety, and potential interactions with standard therapies. Moreover, advancements in drug delivery systems may help overcome challenges related to their bioavailability and stability. In conclusion, HSD and HST represent valuable candidates for the prevention and adjunctive treatment of CRC. Their incorporation into clinical strategies, dietary interventions, or combination therapies holds great promise, but this requires validation through rigorous clinical trials. Future research will be essential to fully harness their therapeutic potential and to integrate these natural agents into personalized medicine approaches for improved CRC management.
